# Treatment of experimental colitis by endometrial regenerative cells through regulation of B lymphocytes in mice

**DOI:** 10.1186/s13287-018-0874-5

**Published:** 2018-05-22

**Authors:** Xiaoxi Xu, Yong Wang, Baoren Zhang, Xu Lan, Shanzheng Lu, Peng Sun, Xiang Li, Ganggang Shi, Yiming Zhao, Hongqiu Han, Caigan Du, Hao Wang

**Affiliations:** 1 Department of General Surgery, Tianjin Medical University General Hospital, 154 Anshan Road, Heping District, Tianjin, 300052 China; 2 Tianjin General Surgery Institute, Tianjin, China; 3 Department of Endocrinology and Metabolism, Tianjin Medical University General Hospital, Tianjin, China; 4 Department of Ultrasound, National Cancer Center/Cancer Hospital, Chinese Academy of Medical Sciences and Peking Union Medical College, Beijing, China; 5 Department of Anorectal Surgery, People’s Hospital of Hunan Province, First Affiliated Hospital of Hunan Normal University, Changsha, Hunan Province People’s Republic of China; 6 Department of General Surgery, Affiliated Hospital of Weifang Medical University, Weifang, Shandong China; 7 Department of Colorectal Surgery, The Second Hospital of Tianjin Medical University, Tianjin, China; 8 Department of Urologic Sciences, the University of British Columbia, Vancouver, British Columbia Canada; 9 Immunity and Infection Research Centre, Vancouver Coastal Health Research Institute, Vancouver, British Columbia Canada

**Keywords:** Ulcerative colitis, Endometrial regenerative cells, B lymphocytes, Immunoregulation, Mice

## Abstract

**Background:**

Endometrial regenerative cells (ERCs), a novel type of mesenchymal-like stem cell derived from menstrual blood, have been recently evaluated as an attractive candidate source in ulcerative colitis (UC); however, the mechanism is not fully understood. The present study was designed to investigate the effects of ERCs, especially on B-cell responses in UC.

**Methods:**

In this study, colitis was induced by administering 3% dextran sodium sulfate (DSS) via free drinking water for 7 days to BALB/c mice. In the treated group, mice were injected intravenously with 1 × 10^6^ ERCs on days 2, 5, and 8 after DSS induction. Therapeutic effects were assessed by monitoring body weight, disease activity, and pathological changes. Subpopulations of lymphocytes were determined by flow cytometry. IgG deposition in the colon was examined by immunohistochemistry staining. Cytokine levels were measured by enzyme-linked immunosorbent assay (ELISA), Western blot, or polymerase chain reaction (PCR) analysis. Adoptive transfer of regulatory B cells (Bregs) into colitis mice was performed.

**Results:**

Here, we demonstrated that ERC treatment prolonged the survival of colitis mice and attenuated disease activity with fewer pathological changes in colon tissue. ERCs decreased the proportion of immature plasma cells in the spleen and IgG deposition in the colon. On the other hand, ERCs increased the production of Bregs and the interleukin (IL)-10 level. Additionally, adoptive transferred Bregs exhibited significant therapeutic effects on colitis mice.

**Conclusions:**

In conclusion, our results unravel the therapeutic role of ERCs on experimental colitis through regulating the B-lymphocyte responses.

## Background

Ulcerative colitis (UC) is a chronic, relapsing, and nonspecific inflammatory disorder of the colon, as shown by an imbalance of the immune system to various factors [1]. It is generally believed that a modified T helper (Th)2-mediated immune response is involved in the pathogenesis of UC, resulting in excessive production of proinflammatory cytokines and the destruction of colonic tissue. However, B cells also play a pathogenic role in UC by producing autoantibodies leading to damage of the intestinal epithelium. The topical deficiency of sIgA and a shift from IgA2 to the less stable IgA1 was reported, as well as a significant increase in IgG-producing cells [2]. Systemically, perinuclear anti-neutrophil antibodies (p-ANCA) and autoantibodies against tropomyosin-5 are found in the peripheral blood of UC patients, and the numbers of immature plasma cells overexpressing chemokines was increased [3]. Unfortunately, salvage therapy with rituximab (anti-CD20 antibody) caused a severe exacerbation of UC [4]. The failure of B cell-depletion therapy was also supported by animal models [5]. Recently, the regulatory role of B cells has been demonstrated in many immune disorders including UC, with a decrease in regulatory B cells (Bregs) [6]. In the TCRα^−/−^ spontaneous colitis model, B cells protected the colon from severe inflammation by generating CD1d upregulated Bregs capable of producing interleukin (IL)-10 [7]. Transferred splenic B cells from parasite infection mice or enteroantigen-pulsed B cells suppress colitis in an IL-10-dependent manner [8, 9]. Furthermore, B cells maintain gut homeostasis by cooperation with regulatory T cells (Tregs) [10] or macrophages [11]. However, the effective strategy that targets the B cell system for maintenance in colitis is still not clear.

Mesenchymal stem cells (MSCs) have been considered as an emerging therapeutic method to treat UC by differentiating into colonic interstitial cells [12] and producing pleiotropic gut trophic factors [13], as well as exerting immunomodulatory effects [14, 15] through inhibiting the responses of macrophages [15] and T cells [16]. According to previous studies, MSCs directly modulate B cell function, such as suppressing terminal differentiation, inhibiting proliferation and antibody production [17–20]. Meanwhile, recent studies have found that MSCs induce the expansion of IL-10-producing Bregs [21]. However, because of the invasive procedures for obtaining MSCs, and their fewer number and limited proliferation capacity due to aging or chronic diseases [22], the application of MSCs from current sources (i.e., the bone marrow, adipose tissue, and umbilical cord) as a cell therapy are restricted.

Endometrial regenerative cells (ERCs) derived from menstrual blood, a novel source of adult stem cells, resemble MSCs with similar phenotypic surface markers, multidifferentiation potential, and immunomodulatory properties [23, 24]. Peculiarly, ERCs possess the unique advantages of a noninvasive procedure for harvesting, their ease of abstraction, and their abundant source. ERCs can be rapidly expanded large scale in vitro with a doubling time of about 20 h while maintaining karyotypic normality up to 68 doublings [25]. Meanwhile, ERCs produce large amounts of growth factors and matrix metalloprotease in favor of tissue repair [23, 25]. We and others have reported the therapeutic effects of ERCs in many disease models, such as critical limb ischemia [23], acute liver injury [26], premature ovarian failure [27], and experimental colitis [28]. Recently, we have demonstrated the immunosuppressive activities against B cell functions in a cardiac transplantation mouse model [29]. However, whether ERCs are capable of regulating the B cell system to protect mice from colitis is unclear. Thus, the present study was undertaken to clarify the immunomodulatory effects of ERCs on B cells in a dextran sodium sulfate (DSS)-induced colitis model.

## Methods

### Animals

Six- to eight-week-old male BALB/c mice weighing 18–22 g (Aoyide Co., Tianjin, China) were used in the present study (24 mice were randomly assigned to the three groups, *n* = 8 per group). All experiments were conducted in accordance with the protocols approved by the Animal Care and Use Committee of Tianjin Medical University (Tianjin, China) according to the Chinese Council on Animal Care guidelines.

### Preparation of ERCs

ERCs were collected and isolated according to a protocol described previously [30, 31]. Briefly, menstrual blood was collected from 20- to 40-year-old healthy women on the first day of the menstruation cycle using Diva cups after informed consent was obtained. Menstrual blood (5 ml) was transferred into a 15-ml centrifuging tube containing 0.2 ml penicillin/streptomycin and 0.2 ml fetal bovine serum (FBS) in 5 ml phosphate-buffered saline (PBS). Mononuclear cells were fractionated by Ficoll-Paque density gradient centrifugation. The interlayer cells were suspended in Dulbecco’s modified Eagle’s medium (DMEM)-F12 supplemented with 10% FBS and 1% penicillin/streptomycin and cultured in a T-25 flank. The culture medium was changed the next day. The adherent cells were then subcultured and passaged twice a week. The morphology of the ERCs was examined by microscopy and the expression of the cell surface markers CD34, CD45, CD90, and CD105 were analyzed by flow cytometry as previously described [25, 28]. Third- and fourth-passage cells were used for treatment.

### Splenic B cell isolation

At necropsy, BALB/c mice spleens were collected and made into a single cell suspension. After magnetic labeling with biotin-conjugated CD19 antibody and anti-biotin microbeads (Miltenyi Biotech, Germany), B cells were isolated by positive selection according to the manufacturer’s protocol.

### Coculture with ERCs

Purified CD19^+^ B cells were cultured in RPMI-1640 medium supplemented with 10% FBS and 1% penicillin/streptomycin at a density of 1 × 10^6^ cells/ml with or without ERCs. Lipopolysaccharide (LPS) was added to the medium at a concentration of 10 μg/ml as a stimulation and, 72 h later, culture supernatant and B cells were collected for further analysis.

To study the effects of ERCs on B-cell proliferation, some purified CD19^+^ cells were stained with carboxyfluorescein succinimidyl ester (CFSE; Invitrogen, Inc., Carlsbad, CA, USA) in PBS/bovine serum albumin (BSA) 0.01% at 37 °C. After 10 min, the reaction was quenched with FBS medium and B cells were collected.

### Colitis model

Colitis was induced in BALB/c mice by administration of 3% (wt/vol) DSS (MP Biochemicals) in free drinking water for 7 days. In the ERC-treated group, ERCs (1 × 10^6^ cells/mouse) were suspended in 200 μl PBS and injected intravenously at days 2, 5, and 8 of DSS administration. In the untreated group, mice were injected with an equal amount of PBS as controls. The body weight was monitored and clinical signs were recorded daily. The Disease Activity Index (DAI) was scored according to the following criteria: a) body weight loss = 0 (no change), 1 (1–5%); 2 (5–10%), 3 (10–20%), and 4 (> 20%); b) stool consistency or diarrhea = 0 (normal), 1 (some soft), 2 (soft), 3 (unformed/mild diarrhea), and 4 (severe watery diarrhea); c) hemoccult positivity and the presence of gross stool blood = 0 (negative fecal occult blood), 1 (negative/positive fecal occult blood), 2 (certain positive fecal occult blood), 3 (visible rectal bleeding), and 4 (severe rectal bleeding). The DAI is the sum of the scores for body weight loss, stool consistency, and gross bleeding, divided by 3. At day 10, mice were sacrificed after euthanization and 5 ml cold PBS was injected into the peritoneal cavity and collected by 3 ml Pasteur tube after the abdomen had been massaged for 3 min. The spleen, mesenteric lymph nodes (MLN), and colon were dissected carefully, and the colon length was measured.

### Flow cytometry analysis of T and B cells

The spleen and MLN were minced gently on ice and filtered through a 40-μm filter. Cells were suspended in staining buffer at a final concentration of 1 × 10^7^/ml and washed twice before blocking with anti-CD16/CD32 Fc-Block (eBioscience, San Diego, CA, USA). Cells were stained with primary monoclonal antibodies at 4 °C in the dark for 30 min. Fluorescein isothiocyanate (FITC)-conjugated anti-CD3, phycoerythrin (PE)-conjugated anti-CD4, and peridinin chlorophyll protein complex (PerCP)-conjugated anti-CD8 were used to detect T cells, and FITC-conjugated anti-CD19, PerCP-Cy5.5-conjugated anti-CD5, allophycocyanin (APC)-conjugated anti-CD1d, PE-conjugated anti-CD83, and PE-conjugated anti-CD138 were used to detect B cells (eBioscience, San Diego, CA, USA) following the manufacturer’s instructions. FITC-conjugated anti-CD4, PE-conjugated anti-CD25, and PE-Cy5-conjugated anti-Foxp3 were used for the detection of Tregs with a Mouse Regulatory T Cell Staining Kit after fixation and permeabilization.

To detect the proportion of IL-10^+^ B cells, intracellular staining was performed. In brief, splenocytes were cultured in complete RPMI-1640 medium and stimulated with Cell Stimulation Cocktail (plus protein transport inhibitors) (eBioscience, San Diego, CA, USA) at a concentration of 2 μl/ml for 5 h. Cells were blocked with anti-CD16/CD32 Fc-Block (eBioscience, San Diego, CA, USA) on ice for 15 min and stained with FITC-conjugated anti-CD19, PerCP-Cy5.5-conjugated anti-CD5, and APC-conjugated anti-CD1d for 20 min. After fixation and permeabilization (Cytofix/Cytoperm kit, BD), intracellular staining was performed on ice with PE-conjugated anti-IL-10 monoclonal antibody for 30 min.

### Enzyme-linked immunosorbent assay (ELISA)

The levels of IL-10 in cell culture supernatants and IL-10, tumor necrosis factor (TNF)-α, IL-1β, IL-6, and IgG in colon tissue homogenous were determined using an ELISA kit (eBioscience, San Diego, CA, USA) according to the manufacturer’s recommended protocol.

### Histology and immunohistochemistry

Colon tissues were fixed in 10% formaldehyde, embedded in paraffin, and sectioned into 5-μm section. Sections were prepared for hematoxylin and eosin (H&E) and immunohistochemistry staining as previously described. IgG deposition in the colon was quantified by immunohistochemical staining with a primary antibody specific for mouse IgG (Abcam). Negative controls were performed using PBS instead of the primary antibody. Sections were examined in a double-blinded fashion by two pathologists.

### Reverse transcription polymerase chain reaction (RT-PCR) and Western blot analysis of IL-10

To determine the splenic IL-10 levels in the perspective of gene transcription, total RNA was extracted from the spleen followed by generation of cDNA and detection with florescent real-time quantitative RT-PCR. The PCR primer was designed as follows: IL-10, upstream 5’-AGAAGCATGGCCCAGAAATCA-3′, downstream 5’-GGCCTTGTAGACACCTTGGT-3′. To examine the protein expression of IL-10 in the spleen, spleens were homogenized in lysis buffer on ice. After centrifugation at 13,000 rpm for 15 min, supernatants were collected to perform Western blotting. A primary antibody specific for mouse IL-10 or β-actin (Santa Cruz Biotechnology) was used in the experiments.

### Adoptive transfer

Purified CD19^+^ B cells from colitis mice treated with or without ERCs were stained with CD1d and CD5 monoclonal antibody. CD1d^hi^CD5^+^ B cells were selected using a flow cytometer (FACSAria, BD) and injected into mice (2 × 10^6^ cells in 250 μl PBS) followed by administration of DSS.

### Statistical analysis

Survival data are presented as mean survival time (MST) and were analyzed using the log-rank test. One-way analysis of variance (ANOVA) and two-tailed, paired *t* tests were used to analyze differences between experimental groups. Differences with *p* values ≤ 0.05 were considered significant.

## Results

### Characterization of ERCs

**Fig. 1:**
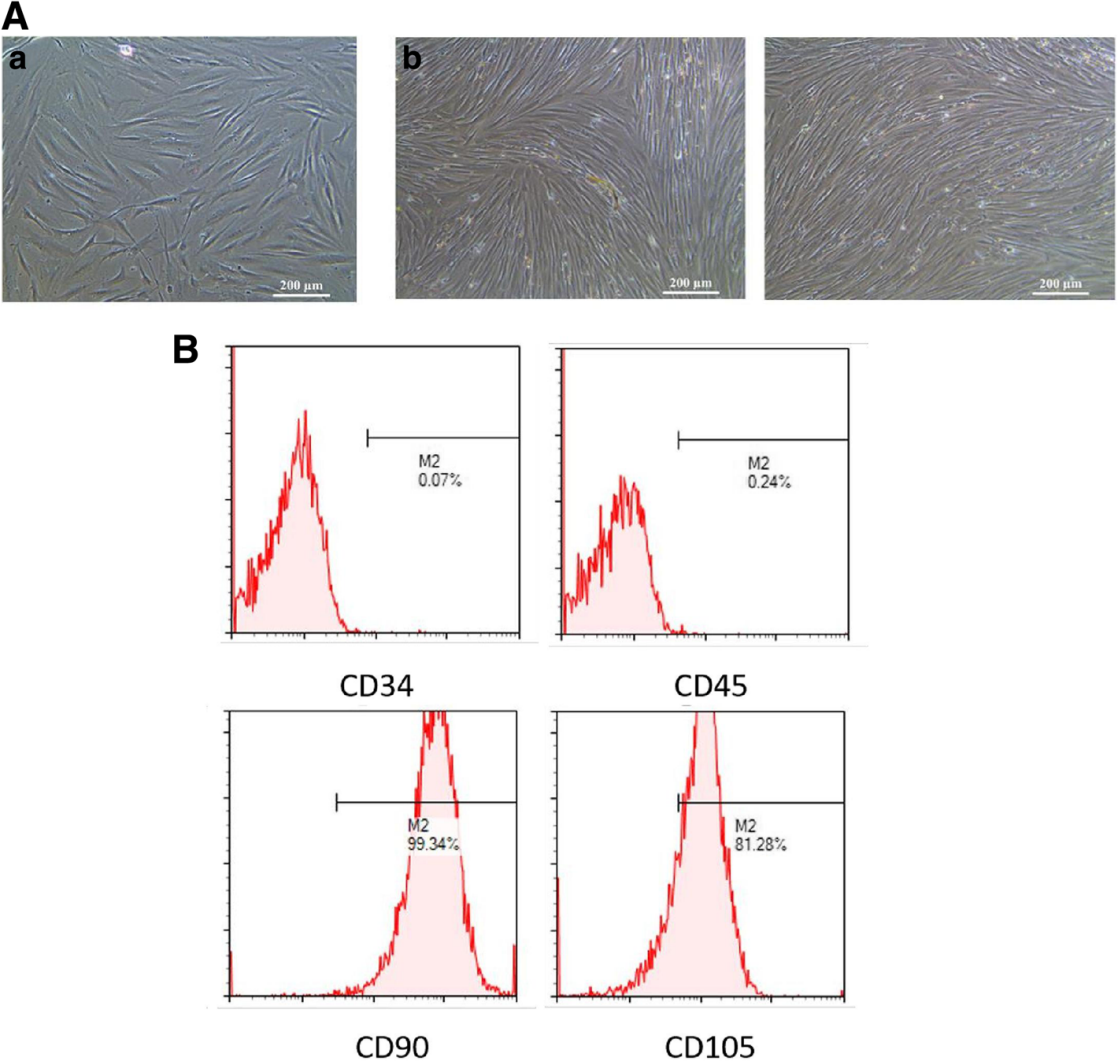
Characterization of ERCs. **A** The morphology of ERCs. **a** P4 passage of ERCs 2 days after subculturing. **b** P4 passage of ERCs 4 days after subculturing. **B** FACS analysis of ERCs using hematopoietic and immunophenotypic markers. Surface expression of CD34, CD45, CD90, and CD105 was detected by flow cytometry. Data shown represent three separate experiments, with similar effects observed in each

ERCs exhibited spindle-shaped, fibroblast-like morphology after passage 3 (Fig. 1A) and colony-forming ability. The doubling time was about 24 h, indicating a high proliferative rate. At passage 4, ERCs were detached and stained with the MSC surface markers CD34, CD45, CD90, and CD105. As reported previously, ERCs demonstrated high expression of CD90 and CD105, while lacking CD34 and CD45 expression (Fig. 1B).

### ERCs attenuated DSS-induced experimental colitis

**Fig. 2:**
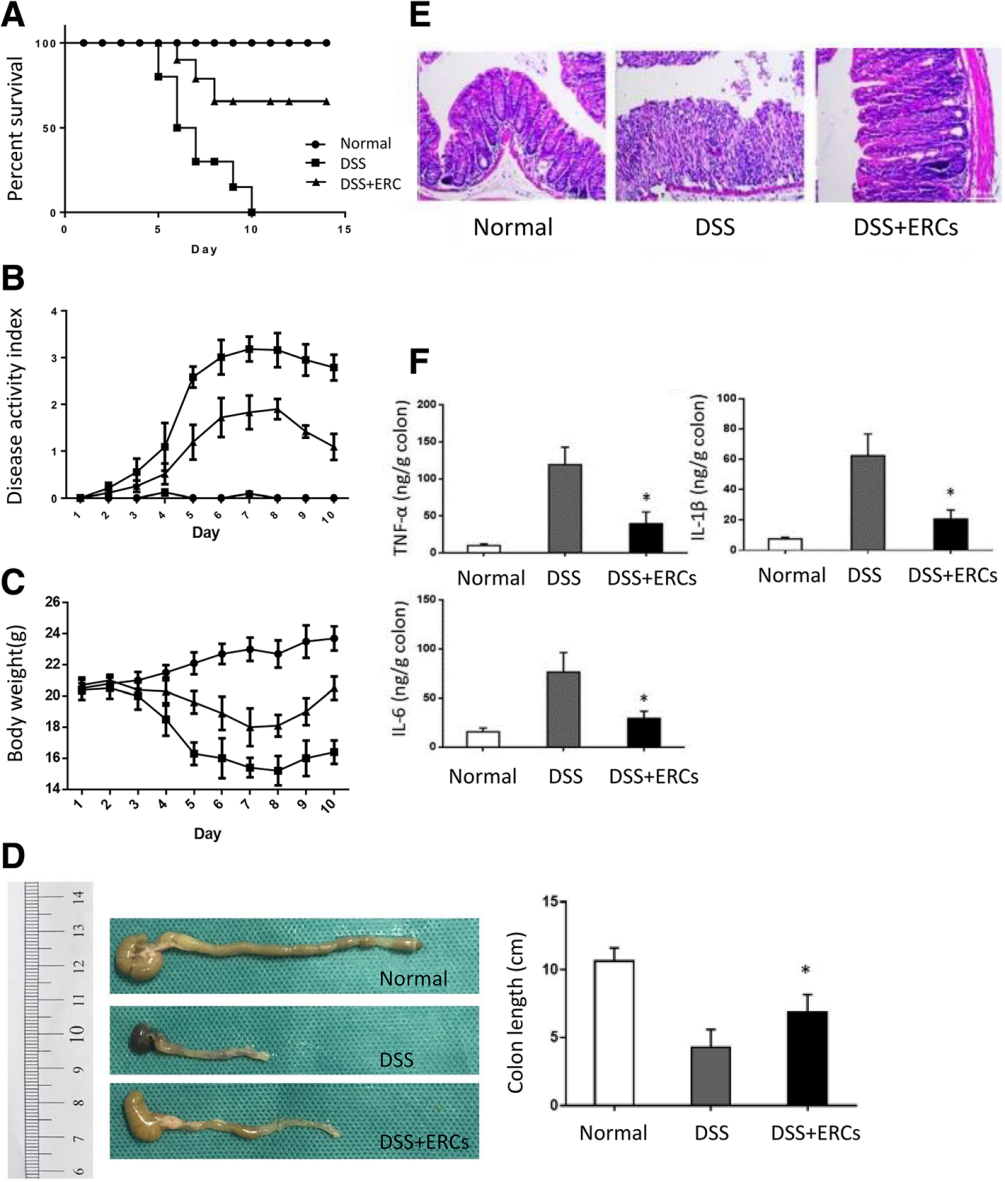
The therapeutic effects of endometrial regenerative cell (ERC) treatment on dextran sodium sulfate (DSS)-induced colitis. BALB/c mice in the ERC-treated group were injected i.v. with ERCs (1 × 10^6^) in 200 μl PBS at days 2, 5, and 8 after DSS induction. Mice in the untreated group were injected i.v. with 200 μl PBS instead. **a** ERCs prolong the survival of DSS-induced colitis mice. Survival rates were monitored daily. *P* value was determined by log-rank survival test. **b**, **c** Body weight, general condition, stool condition, and the appearance of bloody stool were monitored daily. ERCs **b** attenuated the body weight loss and **c** alleviated the clinical severity of DSS-induced colitis mice. *P* value was determined by one-way ANOVA. **d**, **e** Mice were sacrificed at day 10 after DSS induction. Colons were dissected and the distal part was paraffin sectioned and H&E staining was performed. **d** Representative photo showing the colon dissected from mice and **e** the histological sections in each group. **f** ERCs modulated the balance of proinflammatory cytokines in the colon. Colon samples were homogenized and the supernatants were harvested. The concentration of tumor necrosis factor (TNF)-α, interleukin (IL)-1β and IL-6 was measured by ELISA. Graphs represent mean ± SEM of triplicate experiments. *P* value was determined by one-way ANOVA. *P < 0.05

Acute experimental colitis was induced by oral administration of 3% DSS in free drinking water, resulting in severe colitis characterized by body weight loss, bloody diarrhea, and lethargy (Fig. 2a–c). ERC treatment delayed the occurrence of colitis and attenuated its severity, exhibited less body weight loss, and reduced mortality significantly. The general condition, stool consistency, and bloody stool were also improved by ERC treatment (Fig. 2a–c). Consistently, DSS administration lead to the shortening and rigidity of the colon with severe injurious hyperemia and ulceration, which were ameliorated by ERCs (Fig. 2d). Under the microscope, ERCs decreased the pathological changes caused by DSS, including damaged epithelium and crypt structure, glandular disorders, and massive inflammatory cell infiltration into the mucosa and submucosa (Fig. 2e). Meanwhile, the concentration of TNF-α, IL-1β, and IL-6 were analyzed by ELISA. ERC treatment significantly reduced the elevated level of these proinflammatory cytokines caused by DSS administration (Fig. 2f). These results demonstrated that the benefits of ERCs on colitis were probably mediated by anti-inflammatory effects.

### ERCs inhibited Th cells while inducing Tregs during DSS-induced colitis

**Fig. 3:**
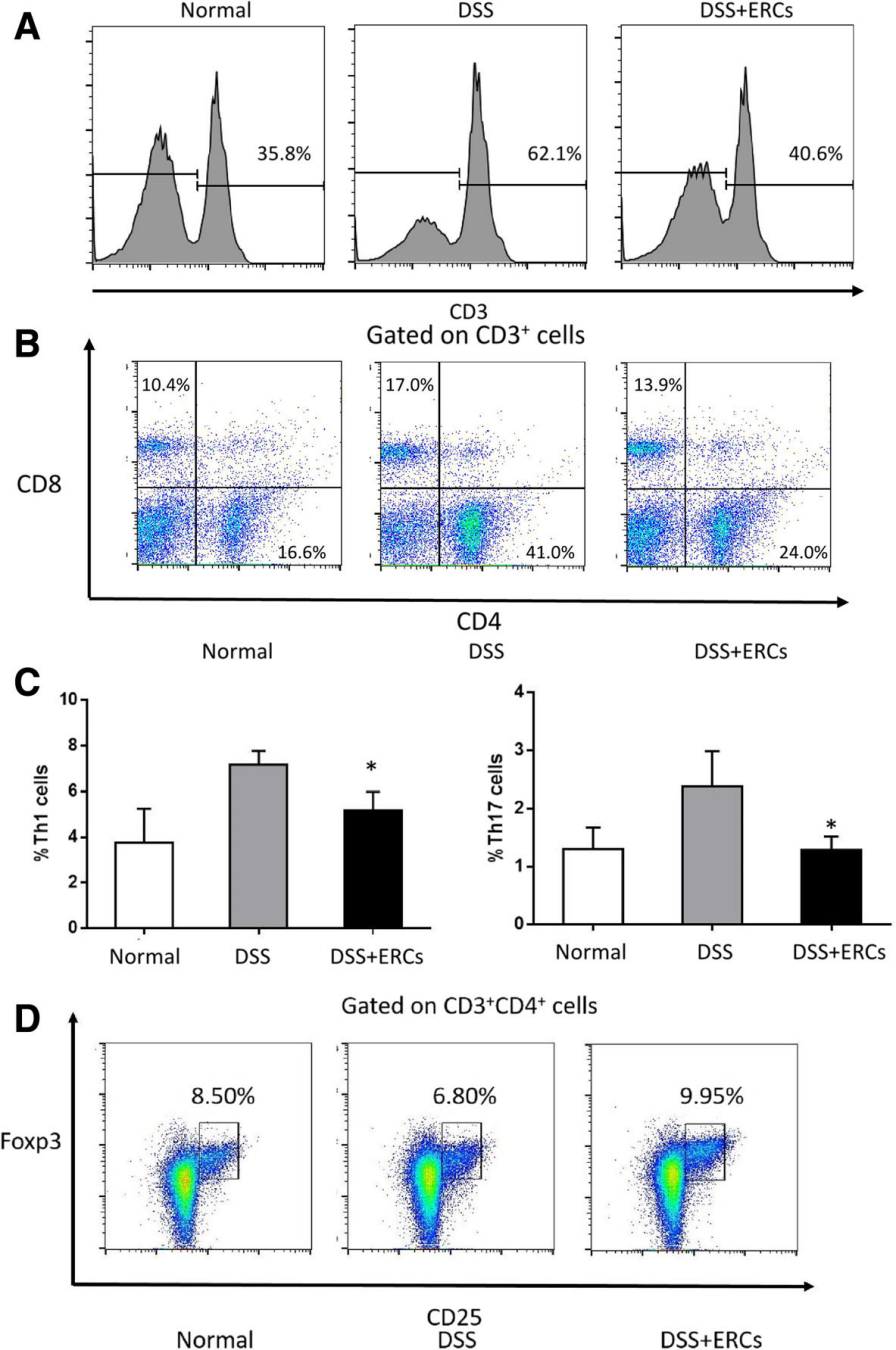
The regulatory effects of endometrial regenerative cell (ERC) treatment on T lymphocytes. The spleen was dissected and made into a single-cell suspension. Cells were stained with fluorescently labeled CD3, CD4, CD8, CD25, IFN-γ, IL-17, and Foxp3, and detected by flow cytometry. The proportion of **a** CD3^+^ T cells in lymphocytes, **b** CD4^+^ and CD8^+^ T cells in CD3^+^ T cells, **c** IFN-γ^+^ and IL-17^+^ T cells in lymphocytes, and **d** CD25^+^Foxp3^+^ Tregs in CD3^+^CD4^+^ T cells was detected by flow cytometry. Graphs represent mean ± SEM of triplicate experiments. *P* value was determined by one-way ANOVA. *P < 0.05. DSS, dextran sodium sulfate; Th, T helper

To explore the immunomodulatory effects of ERCs, flow cytometry was performed to analyze the subpopulation of lymphocytes in the spleen. The proportion of CD3^+^ T cells in lymphocytes as well as CD3^+^CD4^+^ and CD3^+^CD8^+^ T cells was increased in the DSS-treated group, and was inhibited by ERCs (Fig. 3a, b). Importantly, ERCs downregulated the expanded Th1 and Th17 cells in colitis, which are shown by production of the proinflammatory mediators interferon (IFN)-γ and IL-17, respectively, to cause tissue injury (Fig. 3c). On the other hand, ERC treatment elevated the proportion of Tregs in lymphocytes compared with DSS-treated mice, and this was even higher than those from normal mice, which play an important role in the maintenance of homeostasis in colitis (Fig. 3d) [14].

### ERCs inhibited B-cell activation, differentiation, and IgG production in colitis

**Fig. 4:**
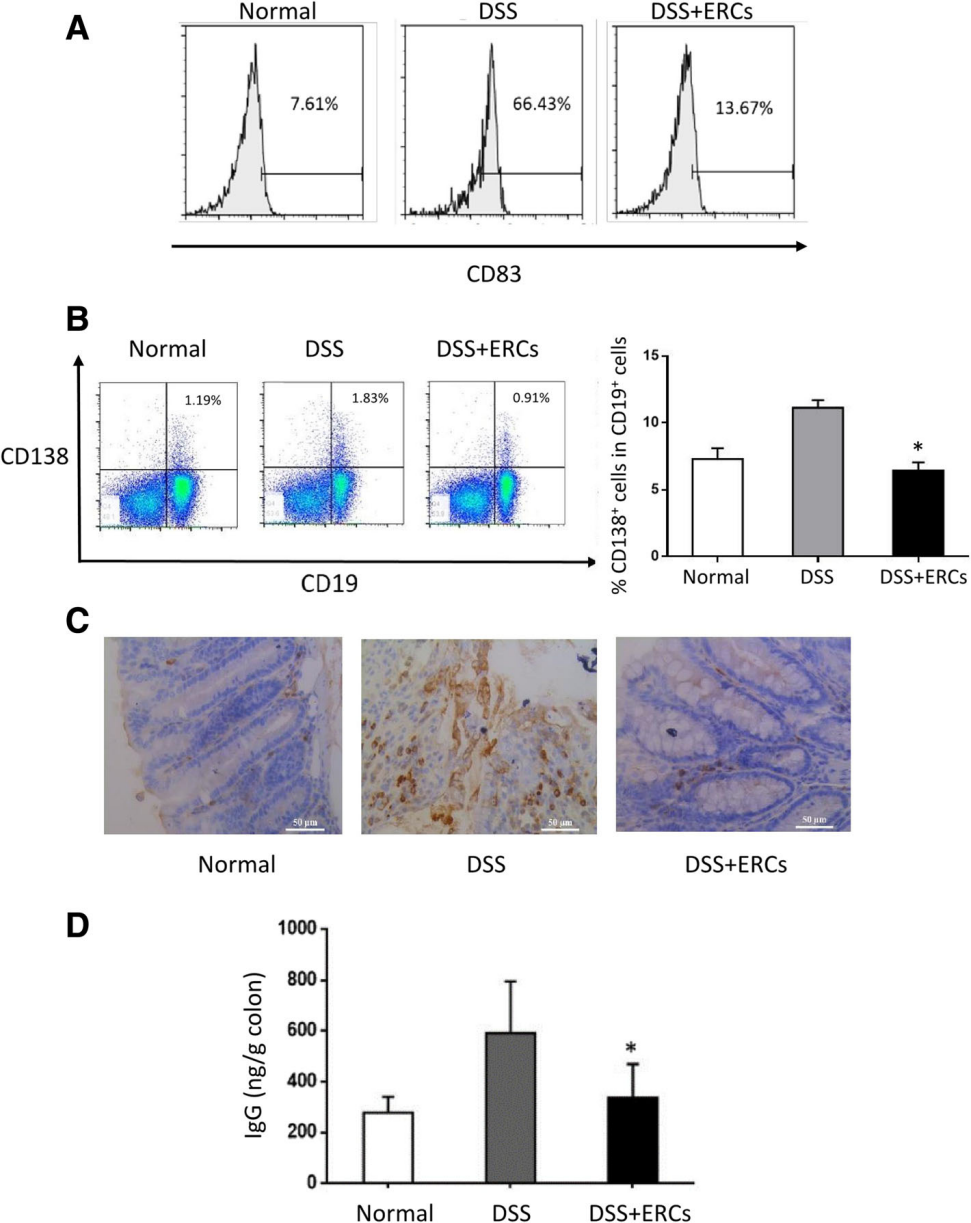
Endometrial regenerative cells (ERCs) inhibited B-cell activation, differentiation, and antibody deposition in colitis mice. The spleen was dissected and made into a single-cell suspension. Cells were stained with fluorescently labeled CD19, CD83, and CD138. **a** The expression of CD83 on CD19^+^ B cells and the proportion of **b** CD19^+^CD138^+^ cells in lymphocytes and CD138^+^ cells in CD19^+^ B cells was detected by flow cytometry. **c** IgG deposition in the colon. Colons were dissected and the distal part was paraffin sectioned with immunohistochemistry staining performed thereafter. Representative photographs of histological sections of colon from normal, untreated, and ERC-treated groups. Antibody deposition was observed by immunohistochemistry specific for IgG (×400). **d** The concentration of IgG in the colon was measured by ELISA. Graphs represent mean ± SEM of triplicate separate experiments. *P* value was determined by one-way ANOVA. *P < 0.05. DSS, dextran sodium sulfate

Although DSS-induced colitis is commonly believed to result from the imbalance of T cells, dysregulation of the B cell system is also involved in the pathogenesis [2]. In the present study, CD83 expression was obviously increased in splenic B cells in DSS-treated mice, indicating the relative active states, which was downregulated by ERC treatment (Fig. 4a). Next, as B cells mainly take part in humoral immunity by differentiating into antibody-producing plasma cells, the proportion of CD138^+^ cells were analyzed. Accordingly, colitis mice exhibited more CD138^+^ immature plasma cells in the spleen, which were reduced by ERC treatment (Fig. 4b). Furthermore, to exclude that these changes were merely caused by the altered ratio of T cells and B cells after DSS induction, the proportion of CD138^+^ cells in CD19^+^ cells was evaluated and showed a similar trend (Fig. 4b). Meanwhile, immunohistochemistry staining suggested that the obvious deposition of IgG in the inflamed colon was also attenuated by ERCs (Fig. 4c), which was confirmed by ELISA assay showing a lower level of IgG in the homogenous colon after ERC treatment (Fig. 4d).

### Treatment with ERCs upregulated the level of IL-10 and promoted the expansion of Bregs

**Fig. 5:**
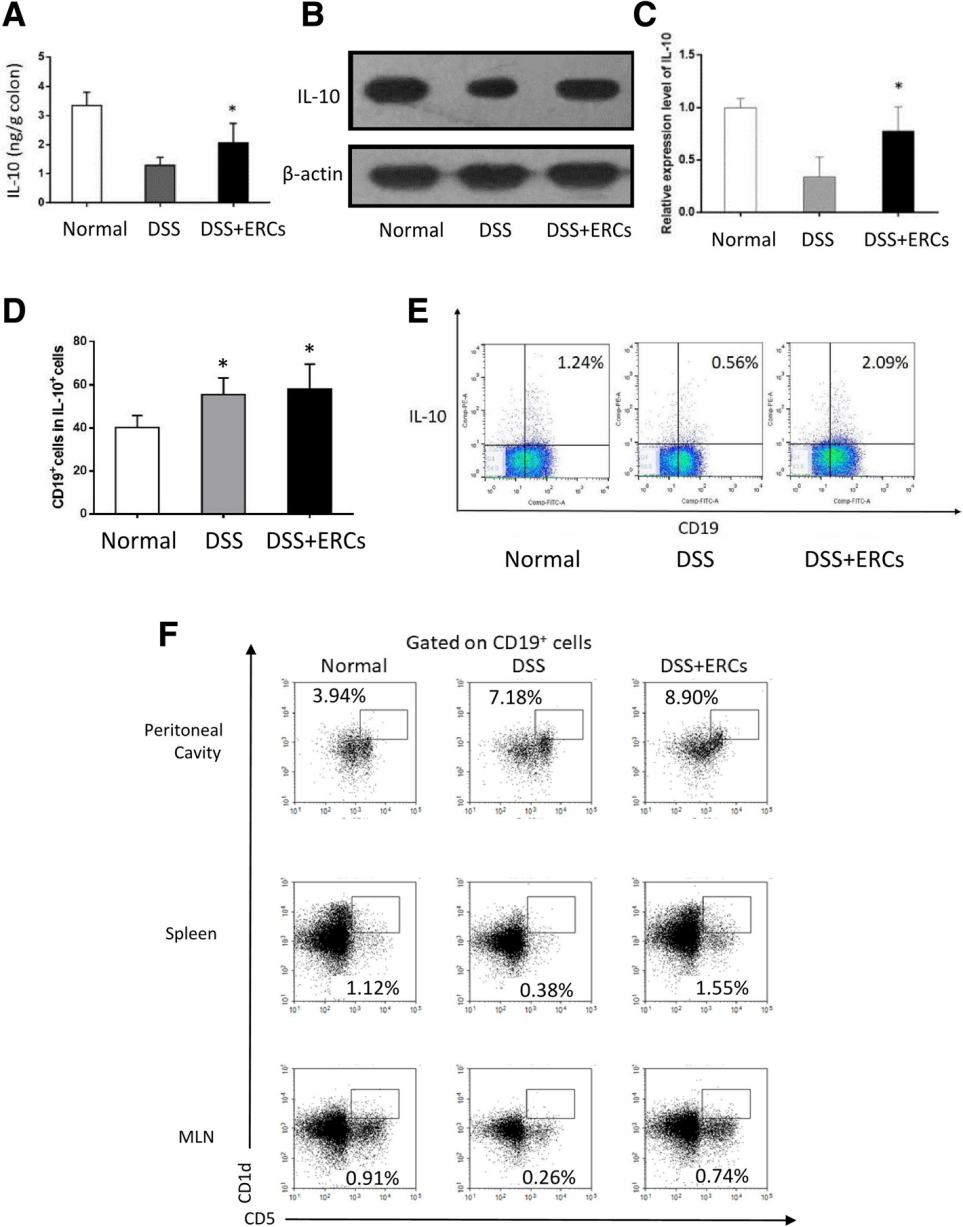
Endometrial regenerative cells (ERCs) upregulated the level of interleukin (IL)-10 and promoted the expansion of Bregs. **a** Colon samples were homogenized and the supernatants were harvested. The concentration of IL-10 in the homogenous colon was measured by ELISA. The **b** protein level and **c** mRNA level of IL-10 in the spleen were examined by Western blot and PCR analysis, respectively. **d** The proportion of CD19^+^ cells in IL-10^+^ cells and **e** IL-10^+^CD19^+^ cells in lymphocytes was detected by flow cytometry. **f** The proportion of CD1d^hi^CD5^+^ Bregs in the spleen, peritoneal cavity, and mesenteric lymph nodes (MLN) was detected by flow cytometry. Graphs represent mean ± SEM of triplicate experiments. *P* value was determined by one-way ANOVA. *P < 0.05. DSS, dextran sodium sulfate

IL-10 is a potent anti-inflammatory cytokine, and the results of IL-10 signaling are present in inflammatory bowel disease (IBD) patients and animal models [32]. In the present study, ERC treatment enhanced the concentration of IL-10 in the colon and spleen (Fig. 5a–c). Based on the results from flow cytometry, we observed that large numbers of IL-10-producing cells were derived from the B-cell pool (Fig. 5d). Thus, we focused on the immunoregulation of ERCs on the balance of the B-cell system in colitis, especially IL-10-competent B cells, and found that ERC-treated mice showed increased IL-10^+^ B cells in the spleen (Fig. 5e).

### ERCs modulated the CD1d^hi^CD5^+^ Bregs in the spleen, MLN, and peritoneal cavity

As CD1d^hi^CD5^+^ B cells are the most well-characterized phenotype of Bregs in mice, we further explored the effects of ERCs on the proportion of CD1d^hi^CD5^+^ B cells in different tissues. As shown in Fig. 5f, the proportion of CD1d^hi^CD5^+^ B cells in the spleen, peritoneal cavity, and MLN was increased by ERC treatment. These results indicated that ERC probably induced the expansion of Bregs to systemically exert protective effects.

### ERCs promoted the production of Bregs in vitro

**Fig. 6:**
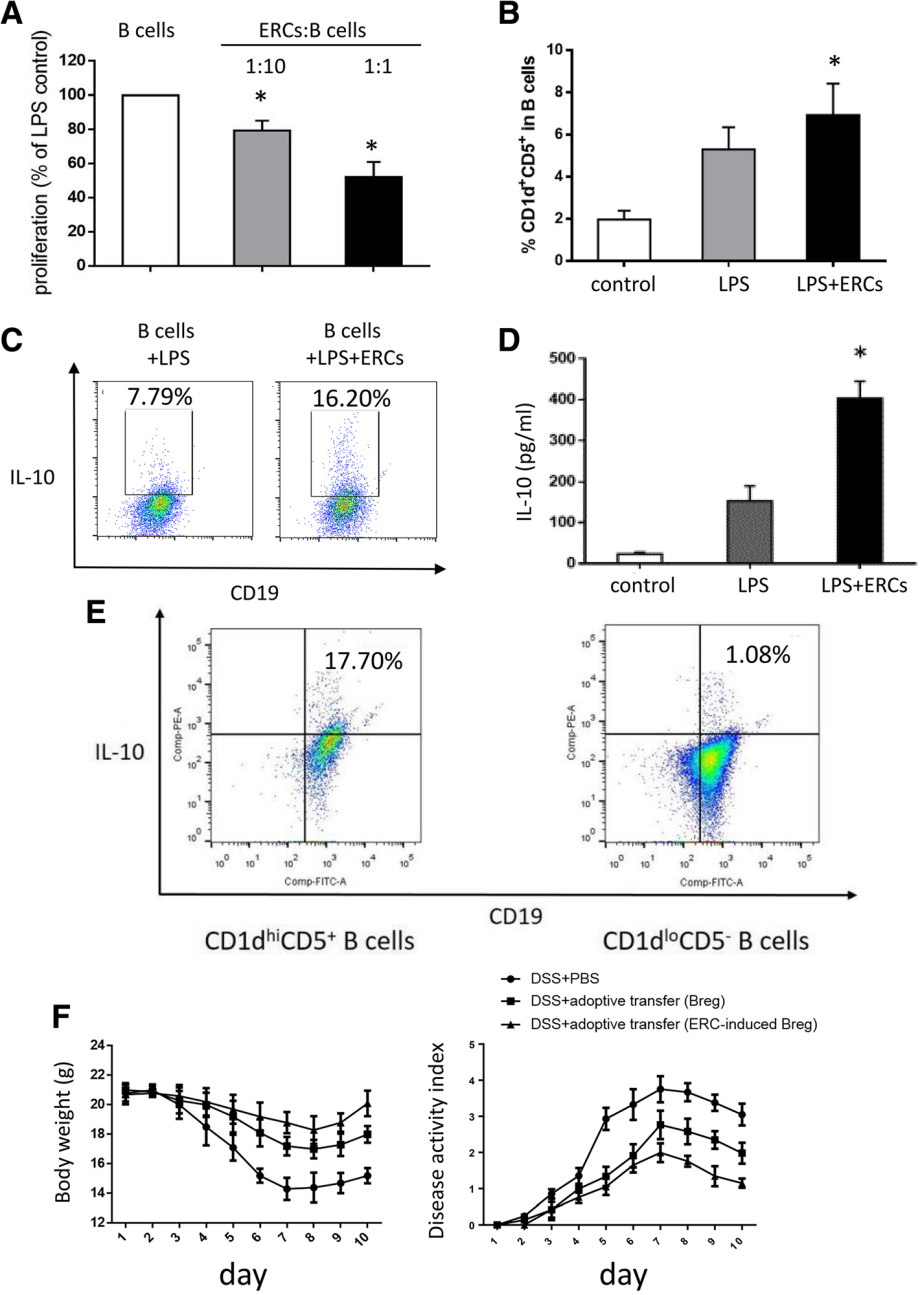
The effects of endometrial regenerative cells (ERCs) on regulatory B cells (Bregs) in vitro and the adoptive transfer of ERC-induced Bregs into colitis mice. Purified recipient B cells (10^6^/well) were stimulated with 10 μg/ml lipopolysaccharide (LPS) for 72 h. **a** CFSE-labeled B cells were harvested thereafter and analyzed by flow cytometry. The proliferation was expressed as a percentage of proliferative B cells versus untreated LPS-stimulated B cells. **b** The proportion of CD1d^hi^CD5^+^ and **c** IL-10^+^ cells in CD19^+^ B cells was measured by flow cytometry. **d** Supernatants were harvested and IL-10 production was measured by ELISA. **e** The expression of IL-10 in CD1d^hi^CD5^+^ and CD1d^low^CD5^−^ B cells from ERC-treated colitis mice. **f** Adoptive transfer of Bregs attenuated body weight loss and disease activity in colitis mice. Graphs represent mean ± SEM of triplicate experiments. *P* value was determined by one-way ANOVA. *P < 0.05. DSS, dextran sodium sulfate; PBS, phosphate-buffered saline

To investigate the direct effects of ERCs on B cells, purified CD19^+^ cells were isolated and cocultured with ERCs. As shown in Fig. 6a, in the presence of LPS as stimulation, ERCs inhibited B-cell proliferation in a dose-dependent manner without inducing apoptosis as we have previously reported (data not shown). On the other hand, ERCs further enhanced the production of CD1d^hi^CD5^+^ B cells (Fig. 6b) and IL-10^+^ B cells (Fig. 6c), with a higher concentration of IL-10 in the culture media (Fig. 6d).

### ERC-induced Bregs ameliorated experimental colitis by an adoptive transfer method

As shown in Fig. 6e, the IL-10 competence of CD1d^hi^CD5^+^ B cells from ERC-treated mice was verified by intracellular staining compared with those of the CD1d^low^CD5^−^ phenotype. Next, to confirm the therapeutic benefits of ERC treatment on colitis, splenic CD1d^hi^CD5^+^ B cells from ERC-treated colitis mice were isolated by flow cytometer and adoptively transferred into mice before DSS administration. We found that ERC-induced Bregs exhibited obvious therapeutic effects on colitis and attenuated the body weight loss of colitis mice (Fig. 6f).

## Discussion

As UC is mainly mediated by an atypical Th2 immune reaction, the imbalance of the B-cell system also contributes to the occurrence and aggravation of UC. Autoantibody production and deposition resulted in the destruction of intestinal epithelium and tissue injury [2]. On the other hand, IL-10-producing Bregs, which are believed to serve as an immune modulator, were impaired in UC patients [6]. MSCs modulated B-cell function both in vivo and in vitro; however, several shortcomings limit the application of MSCs [17, 33]. ERCs are a novel type of adult MSC derived from menstrual blood; they share similar surface markers and immunomodulatory properties to MSCs, but possess the specific advantages of a noninvasive procedure of harvesting, ease of extraction, and an abundant source [23, 24]. In this study, we investigated the therapeutic effects of regulating B cells in DSS-induced colitis. ERCs inhibited B-cell activation, differentiation, and antibody deposition in the colon, while they induced Breg expansion and promoted IL-10 generation. Additionally, adoptive transferred Bregs from ERC-treated mice alleviated colitis, indicating the therapeutic role of ERCs on experimental colitis through regulating the B-lymphocyte responses.

In recent years, MSCs have been considered as an attractive tool in the treatment of UC. In addition to their multidifferentiation potential [12] and trophic factor secretion [13], MSCs exert immunomodulatory effects on macrophages, dendritic cells, and T cells to attenuate colitis [14–16]. Of note, the immunomodulatory effect is not MHC-restricted, since colitis mice can receive a benefit from xenogeneic MSCs as well as syngeneic and allogeneic MSCs [16]. Human MSCs from different sources [14, 34] are well tolerated by mice and have effects on other diseases [35], underling the possibility of the application of xenogeneic ERCs.

As reported previously, several types of autoantibodies have been detected in the serum of UC patients, such as xANCAs and hTM5-specific IgG [36], and local infiltration of IgG-producing plasma cells in the inflamed mucosa increases [2]. We have demonstrated ERC-mediated inhibitory effects on B-cell proliferation and antibody production in a cardiac transplantation model [37]. In this study, ERCs inhibited the overactivated B cells of colitis mice. Meanwhile, since the activation surface marker CD83 is the costimulatory molecule, ERCs may suppress the antigen-presenting function of B cells and subsequent T-cell response indirectly.

ERC treatment downregulated local IgG deposition and the proportion of splenic immature plasma cells, suggested that ERCs may protect the colon from the humoral response directly by means of inhibiting de novo production of autoantibody or B-cell terminal differentiation and chemotaxis to the colon in a similar manner to MSCs. This is supported by previous findings that human bone marrow-derived MSCs downregulated the expression of chemokines on B cells, such as CXCR4 and CXCR5, which are important for B-cell migration and positioning in secondary lymphoid organs such as the spleen [20].

However, B-cell depletion therapy with anti-CD20 monoclonal antibody results in the exacerbation of colitis [5]. In contrast, ERCs could functionally inhibit B cells without inducing apoptosis [37], but further promote the expansion of IL-10-producing Bregs both in vitro and in vivo. Bregs are capable of skewing the differentiation of Tregs and maintaining a Treg pool [38], the role of which has been extensively investigated in many immune-related diseases such as systemic lupus erythematosus (SLE), experimental autoimmune encephalomyelitis (EAE), and arthritis [39]. The suppressive effects of B cells in colitis have been demonstrated by TCR-α^−/−^ × Igμ^−/−^ mice lacking B cells spontaneously developing more severe colitis than TCR-α^−/−^ mice [40] and adoptive transfer of IL-10-producing CD1d^+^CD5^+^ Bregs inhibiting the DSS-induced intestinal injury in a mouse model [41].

IL-10 competence is the key marker of Bregs. IL-10-deficient mice are more susceptible to chronic colitis [42] while IL-10-transgenic spleen cells prevent colitis induction [43]. IL-10 maintains the expression of Foxp3 on Tregs [44], an important source of IL-10 in colitis mice [45]. In this study, we found the upregulation of IL-10 and Tregs after ERC treatment in colitis mice. Thus, it is tempting to think that ERC may induce the production of IL-10-producing B cells, which results in and from the expansion of Tregs to form a complex loop to regulate the gut homeostasis and suppress colitis, as stated previously [10].

Recently, the induction of Bregs by MSCs has been reported. Qin et al. found that MSCs derived from mouse bone marrow induce Bregs via the stromal cell-derived factor (SDF)-1α–CXCR7 axis [21]. Interestingly, as we reported recently, SDF-1α also mediates the induction of regulatory immune cells by ERCs, including CD1d^+^CD5^+^ Bregs [29]. In the present study, we found that ERCs increased the fraction of CD1d^+^CD5^+^ Bregs in the spleen and MLN, as well as in the peritoneal cavity. ERCs may migrate to these lymphoid organs to induce the expansion of Bregs directly, or may promote the circulation of Bregs among gut-associated lymphoid tissues. To date, it has been suggested that stem cells might contribute as tropic suppliers by stimulating tissue repair through the secretion of paracrine factors. According to Legaki et al., conditioned medium derived from the spindle-shaped amniotic fluid MSCs is able to ameliorate DSS-induced colitis [46], underlining the possibility that ERCs may modulate the immune system in colitis mice in a similar manner. More in-depth studies are warranted to clarify how ERCs are involved in this procedure.

To examine the direct effect of Bregs in ERC-based therapy, we isolated CD1d^+^CD5^+^ Bregs from the spleen of ERC-treated colitis mice to perform adoptive transfer, which is an important IL-10 source. Interestingly, it is likely that ERC-induced Bregs exhibited obvious therapeutic effects on colitis (being even more effective than those from colitis mice without ERC treatment), indicating that the upregulation of Bregs was not a simple response to inflammation or the result of Treg reactions, but was also part of the immunomodulatory effects of ERC treatment.

## Conclusions

In summary, this study for the first time demonstrates that ERCs regulate the balance of the B-cell response in experimental colitis mice. ERCs inhibited splenic B-cell differentiation and reduced the deposition of IgG antibodies in the intestinal tissue of colitis mice. On the other hand, ERCs increased the production of IL-10 and the proportion of CD1d^+^CD5^+^ Bregs from colitis mice, an important source of IL-10. ERC-pulsed Bregs effectively alleviated the symptoms of colitis and promoted intestinal recovery, indicating that ERCs may exert protective effects through Bregs. Thus, it is possible that ERC-mediated B-cell regulation, at least in part, contributes to protection of the colon from severe inflammation in this preclinical model. Again, ERCs with their unique features of ease of collection, a relatively unlimited source, the immunomodulatory effect, and hypoimmunogenicity, as well as the lack of tumorigenesis or tumor acceleration, could make them an attractive novel source of stem cells for cytotherapy for the prevention and/or treatment of ulcerative colitis.

## Abbreviations

Breg: Regulatory B cell
DAI: Disease activity index
DSS: Dextran sodium sulfate
ERC: Endometrial regenerative cell
IBD: Inflammatory bowel disease
LPS: Lipopolysaccharides
MSC: Mesenchymal stem cell
Treg: Regulatory T cell
UC: Ulcerative colitis
